# Sensitivity and Diagnostic Yield of the First SARS-CoV-2 Nucleic Acid Amplification Test Performed for Patients Presenting to the Hospital

**DOI:** 10.1001/jamanetworkopen.2022.36288

**Published:** 2022-10-12

**Authors:** Corinne M. Hohl, Jeffrey P. Hau, Samuel Vaillancourt, Jennifer Grant, Steven C. Brooks, Laurie J. Morrison, Jeffrey J. Perry, Rhonda J. Rosychuk

**Affiliations:** 1 Department of Emergency Medicine, Faculty of Medicine, University of British Columbia, Vancouver, British Columbia, Canada; 2 Centre for Clinical Epidemiology and Evaluation, Vancouver Coastal Health Research Institute, Vancouver, British Columbia, Canada; 3 Department of Emergency Medicine, Li Ka Shing Knowledge Institute, St Michael’s Hospital, Unity Health Toronto, Toronto, Ontario, Canada; 4 Division of Emergency Medicine, Department of Medicine, Faculty of Medicine, University of Toronto, Toronto, Ontario, Canada; 5 Division of Medical Microbiology and Vancouver Coastal Health, Vancouver, British Columbia, Canada; 6 Division of Infectious Diseases, University of British Columbia, Vancouver, British Columbia, Canada; 7 Department of Emergency Medicine, Faculty of Health Sciences, Queen’s University, Kingston, Ontario, Canada; 8 Department of Emergency Medicine, St Michael’s Hospital, Unity Health Toronto, Toronto, Ontario, Canada; 9 Department of Emergency Medicine, University of Ottawa, Ottawa, Ontario, Canada; 10 Ottawa Hospital Research Institute, Ottawa, Ontario, Canada; 11 Department of Pediatrics, University of Alberta, Edmonton, Alberta, Canada

## Abstract

**Importance:**

Early and accurate diagnostic testing for SARS-CoV-2 is essential to initiate appropriate treatment and infection control and prevention measures among patients presenting to the hospital.

**Objective:**

To evaluate the diagnostic sensitivity of the SARS-CoV-2 nucleic acid amplification test (NAAT) performed within 24 hours of arrival to the emergency department among a nationally representative sample of patients.

**Design, Setting, and Participants:**

This diagnostic study was conducted at 47 hospitals across 7 provinces in Canada participating in the Canadian COVID-19 Rapid Response Emergency Department Network among consecutive eligible patients presenting to a participating emergency department who were tested for SARS-CoV-2 from March 1, 2020, to December 31, 2021. Patients not tested within 24 hours of arrival and those presenting with a positive result from a test performed in the community were excluded.

**Main Outcomes and Measures:**

The primary outcome was a positive result from the SARS-CoV-2 NAAT. Outcome measures were the diagnostic sensitivity and yield of the SARS-CoV-2 NAAT.

**Results:**

Of 132 760 eligible patients (66 433 women [50.0%]; median age, 57 years [IQR, 37-74 years]), 17 174 (12.9%) tested positive for SARS-CoV-2 within 14 days of their first NAAT. The diagnostic sensitivity of the SARS-CoV-2 NAAT was 96.2% (17 070 of 17 740 [95% CI, 95.9%-96.4%]) among all of the tests performed. Estimates ranged from a high of 97.7% (1710 of 1751 [95% CI, 96.8%-98.3%]) on day 2 of symptoms to a low of 90.4% (170 of 188 [95% CI, 85.3%-94.2%]) on day 11 of symptoms among patients presenting with COVID-19 symptoms. Among patients reporting COVID-19 symptoms, the sensitivity of the SARS-CoV-2 NAAT was 97.1% (11 870 of 12 225 [95% CI, 96.7%-97.3%]) compared with 87.6% (812 of 927 [95% CI, 85.2%-89.6%]) among patients without COVID-19 symptoms. The diagnostic yield of the SARS-CoV-2 NAAT was 12.0% (18 985 of 158 004 [95% CI, 11.8%-12.2%]) and varied from a high of 20.0% (445 of 2229 [95% CI, 18.3%-21.6%]) among patients tested on day 10 after symptom onset to a low of 8.1% (1686 of 20 719 [95% CI, 7.7%-8.5%]) among patients presenting within the first 24 hours of symptom onset.

**Conclusions and Relevance:**

This study suggests that the diagnostic sensitivity was high for the first SARS-CoV-2 NAAT performed in the hospital and did not vary significantly by symptom duration. Repeated testing of patients with negative test results should be avoided unless their pretest probability of disease is high.

## Key Points

**Question:**

What is the diagnostic sensitivity of the SARS-CoV-2 nucleic acid amplification test (NAAT) by date of symptom onset among patients presenting to the hospital?

**Findings:**

In this diagnostic study, the sensitivity of the first SARS-CoV-2 NAAT performed in the hospital was high within 14 days of symptom onset. Diagnostic yield was highest among patients presenting on day 10 of illness.

**Meaning:**

The high diagnostic sensitivity of the NAAT in this study suggests that 1 negative test result can rule out SARS-CoV-2 infection among patients in the emergency department; only patients with high clinical pretest probability of disease should undergo repeated testing.

## Teaser

This study evaluates the diagnostic sensitivity of the SARS-CoV-2 nucleic acid amplification test performed within 24 hours of arrival to the emergency department among a nationally representative sample of patients.

## Introduction

Early and accurate diagnosis of COVID-19 is key to making appropriate treatment decisions for vulnerable patients and ensuring successful public health measures to mitigate the pandemic. Nucleic acid amplification tests (NAATs) are considered the criterion standard for detecting SARS-CoV-2.^1,2^ However, obtaining a positive test result for an individual infected with SARS-CoV-2 is contingent on sampling technique and the presence of a sufficient amount of the virus at the anatomical site of sampling. Studies from the early pandemic including mostly hospitalized patients with high positivity rates showed that changes in viral shedding over time were associated with decreasing sensitivity of diagnostic tests and increasing false-negative test results within days of symptom onset.^3,4,5,6,7^ False-negative test results have the potential to falsely reassure patients and their caregivers and may be associated with the spread of the disease if patients are not isolated. In contrast, unnecessary widespread repeated testing in areas of low prevalence strains testing resources, may delay removal of patient isolation measures, and may result in higher rates of false-positive NAAT results, which may harm uninfected individuals if they test positive and cannot work or if they are isolated with infected patients.^8^

Gaining a better understanding of the clinical diagnostic test performance of NAATs in association with time since symptom onset and disease prevalence may help clinicians correctly interpret diagnostic test results, develop rational testing strategies, and avoid unnecessary repeated testing. One study illustrated the importance of this process by modeling the association of a late testing strategy with false-negative test results and estimated a quadrupling of false-negative test results compared with an early testing strategy.^7^ Correct interpretation of diagnostic test results can guide treatment, retesting decisions, and return-to-work recommendations.^9,10^

Our aim was to evaluate the clinical sensitivity and diagnostic yield of SARS-CoV-2 NAATs among a national cohort of patients presenting to emergency departments across pandemic waves. Secondary aims included evaluating the sensitivity and diagnostic yield of NAATs by symptom duration and identifying risk factors for false-negative test results. Owing to the lack of an independent reference standard against which NAAT results could be evaluated, we compared the result of the initial NAAT performed in the hospital with results of repeated tests performed within 14 days.

## Methods

### Design and Setting

This diagnostic test evaluation study used data from the Canadian COVID-19 Emergency Department Rapid Response Network (CCEDRRN),^11,12^ which is a multicenter, pan-Canadian registry that retrospectively enrolled patients presenting to emergency departments with suspected COVID-19 from March 1, 2020, onward to enable high-quality observational studies.^12,13,14^ Information about the registry, including data collection methods, data validation, and a list of participating sites, has been published.^11^ The study protocol was reviewed and approved by the University of British Columbia Ethics Board with a waiver for informed consent for patient enrollment because patient data were deidentified, allowing us to capture data on consecutive eligible patients, including populations commonly excluded from trials. This study followed the methodological and reporting recommendations outlined in the Standards for Reporting of Diagnostic Accuracy (STARD) reporting guideline.^15^

### Participants

This study included patients from CCEDRRN sites that were able to demonstrate enrollment of 99% or more of eligible patients to minimize selection bias. We included consecutive patients who were tested for SARS-CoV-2 at 47 sites across 7 provinces in Canada starting March 1, 2020, and ending December 31, 2021 (eTable 1 in Supplement 1). We excluded patients who were not tested using a SARS-CoV-2 NAAT while they were in the emergency department or, if admitted, within 24 hours of emergency department arrival. For time-dependent analyses of sensitivity and diagnostic yield by symptom duration, we excluded patients without a reported date of symptom onset and those with symptoms for more than 14 days. For patients with multiple emergency department encounters, we used their first encounter for this analysis. eTable 1 in Supplement 1 lists the number of patients recruited by site and data collection period who contributed to this analysis.

### Data Collection

The CCEDRRN registry collects data on prespecified demographic and social variables, vital signs, symptoms, and comorbid conditions (derived from the International Severe Acute Respiratory and Emerging Infection Consortium reporting form)^16^; exposure risk variables; hospital laboratory test results; diagnostic imaging results; health resource use; and patient outcomes. Trained research assistants abstracted data at each site using electronic medical records or manually reviewed electronic or paper medical records depending on site-specific documentation practices. Research assistants were blinded to the objectives of this analysis.

A previous study evaluated the reliability of health record data by comparing key clinical variables abstracted retrospectively from the health records with prospective data obtained from a sample of patients and found that most variables had high correlation.^11^ The central coordinating office conducted regular data quality checks and verified extreme and outlying values at each participating site to ensure high-quality data. A national coordinator reviewed study logs at each site to ensure that 99% or more of consecutive eligible patients were captured.

### Outcome

The primary outcome was a positive SARS-CoV-2 NAAT result. Outcome measures were the diagnostic sensitivity and yield of the SARS-CoV-2 NAAT.

### Definitions

We defined patients with suspected COVID-19 as those who were tested for SARS-CoV-2. We defined a false-negative test result as a negative SARS-CoV-2 NAAT result followed by a positive SARS-CoV-2 NAAT result within 14 days of the negative test result, reflecting the upper limit of the SARS-CoV-2 incubation period.^6^ We considered the subsequent positive test result of repeatedly tested patients as the criterion standard given the high analytical specificity of the NAAT estimated at 98.9% or more, and we considered all positive NAAT results to be true analytic positive test results.^17,18,19^ We used the standard test in each clinical laboratory for the diagnosis of COVID-19 as the reference standard. We defined COVID-19 symptoms as any one of the following: cough; shortness of breath; fever; chills; headache; nausea or vomiting; diarrhea; dysgeusia or anosmia; sputum production; hemoptysis; sore throat; runny nose; chest pain, discomfort, or tightness; myalgia; and fatigue. We defined severe COVID-19 according to World Health Organization criteria.^20^ For adults, criteria for severe COVID-19 included an oxygen saturation of less than 90% while breathing room air, a respiratory rate of more than 30 breaths per minute, or documented signs of severe respiratory distress while in the emergency department.

### Sample Size and Precision

This study did not aim to reject a null hypothesis. Therefore, the sample size was calculated based on the desired precision of the estimated sensitivity of the diagnostic test. We estimated requiring 1000 patients per stratum with a positive SARS-CoV-2 test result to estimate a sensitivity of 90% within a 95% CI of ±3%. At the time of the data cutoff, we believed that we would exceed this number of patients for most time strata. Because this was an observational study based on available data, we used all available data.

### Statistical Analysis

We used descriptive statistics to describe the patient population with measures of variance appropriate to the data distribution. We allowed all NAATs performed for a given patient to be entered into the analysis of sensitivity and diagnostic yield until the first positive SARS-CoV-2 NAAT result was obtained. Subsequent positive test results were not included in the analysis. Our primary analysis was the sensitivity or true-positive rate of SARS-CoV-2 NAATs. Sensitivity was defined as true-positive NAAT results divided the false-negative results plus true-positive NAAT results. We then calculated sensitivity by time interval since symptom onset, excluding patients who did not report a date of symptom onset within 14 days. We performed sensitivity analyses by comparing the true-positive rate among patients who reported COVID-19 symptoms in the emergency department with patients without COVID-19 symptoms and also with patients who were health care workers or from long-term care facilities because they may have been tested more frequently and for different testing indications than the general public. We calculated the diagnostic yield as all positive NAAT results divided by all NAATs performed and by time interval of symptom duration, excluding patients who did not report a date of symptom onset within 14 days. We presented all proportions with 95% CIs.

We used logistic regression, with a random effect for study sites for our univariate and multivariate analyses, to identify risk factors for initial false-negative test results. We adjusted for age at presentation, sex assigned at birth, time interval since symptom onset, comorbid conditions, symptoms at presentation, respiratory variables, and the 7-day mean incident COVID-19 case count in the patient’s health region. The latter variable allowed us to adjust for the patient’s community incidence of COVID-19 at the time of their emergency department presentation. We constructed the variable by computing each patient’s 7-day moving mean incident COVID-19 cases per 100 000 population using each patient’s forward sortation area of their postal code of residence and the date of their emergency department admission.^13^ We fit continuous variables, such as the 7-day moving mean incident COVID-19 cases and the time interval since symptom onset, with restricted cubic splines with 3 knots into our logistic regression models. We ensured a minimum of 10 outcomes (false-negative test results) per covariate to avoid overfitting in our univariate analyses. We obtained the multivariate model by excluding variables until all remaining variables were found to be associated with false-negative test results at a 2-sided *P* < .05 threshold. We conducted analyses using Stata, version 16.1 (StataCorp LLC).

## Results

**Figure 1.**
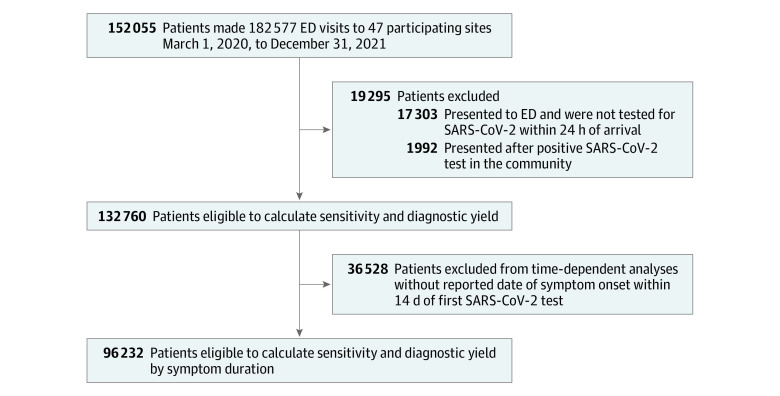
Patient Flow Diagram ED indicates emergency department.

Of 132 760 eligible patients (66 433 women [50.0%]; median age, 57 years [IQR, 37-74 years]) who presented to 47 emergency departments, 96 232 reported a date of symptom onset within 14 days (Figure 1; Table 1; eTable 1 in Supplement 1). The mean (SD) number of days between symptom onset and the first NAAT was 4.4 (3.6) days. The most common presenting symptoms were cough, fever, and chills. Among 17 174 patients (12.9%) with a positive SARS-CoV-2 test result during their hospital visit, most arrived from home. The most common comorbid conditions among patients with COVID-19 were hypertension (5116 [29.8%]) and diabetes (2901 [16.9%]). The proportion of patients who were admitted to the hospital, treated with corticosteroids, or experienced severe COVID-19 increased as the number of days between symptom onset and in-hospital testing increased, while the proportion of patients who died in the hospital or were intubated remained relatively constant over time (eTables 2 and 3 and eFigure in Supplement 1).

**Table 1.** Patient Characteristics by COVID-19 Status

Characteristic	Patients, No. (%)	
	Negative test result (n = 115 586)	Positive test result (n = 17 174)
Age, median (IQR), y	58.0 (37.0-75.0)	54.0 (36.0-71.0)
Sex		
Female	58 460 (50.6)	7973 (46.4)
Male	57 090 (49.4)	9197 (53.5)
Intersex	34 (0.03)	<5
Pandemic wave		
Wave 1 (March 1 to June 30, 2020)	29 433 (25.5)	2073 (12.1)
Wave 2 (July 1, 2020, to February 28, 2021)	41 197 (35.6)	7839 (45.6)
Wave 3 (March 1 to July 14, 2021)	30 797 (26.6)	4700 (27.4)
Wave 4 (July 15 to December 31, 2021)	14 159 (12.3)	2562 (14.9)
Arrival to ED from		
Home	102 664 (88.8)	15 489 (90.2)
Long-term care, rehabilitation facility, or corrections	5899 (5.1)	837 (4.9)
No fixed address, shelter, or single-room occupancy	3841 (3.3)	539 (3.1)
Interhospital transfer	1790 (1.6)	97 (0.6)
Other	1294 (1.1)	181 (1.1)
Risk for infection		
Health care worker	1888 (1.6)	336 (2.0)
Household or caregiver contact	1647 (1.4)	2907 (16.9)
Institutional exposure (long-term care or corrections)	7561 (6.5)	1178 (6.9)
Microbiology laboratory contact	28 (0.02)	<5
Travel	1638 (1.4)	385 (2.2)
No documented risk for infection	95 789 (82.9)	10 940 (63.7)
Arrival vital signs, median (IQR)		
Body temperature, °C^a^	36.7 (36.3-37.0)	36.9 (36.5-37.5)
Heart rate, beats/min^a^	90.0 (77.0-105.0)	94.0 (82.0-108.0)
Oxygen saturation, %^a^	97.0 (96.0-99.0)	97.0 (95.0-98.0)
Respiratory rate, breaths/min^a^	18.0 (16.0-20.0)	18.0 (18.0-22.0)
Systolic blood pressure, mm Hg^a^	113.0 (119.9-150.0)	129.0 (116.0-144.0)
Common comorbid conditions		
Active malignant neoplasm	9482 (8.2)	696 (4.1)
Asthma	7739 (6.7)	1086 (6.3)
Atrial fibrillation	8958 (7.8)	852 (5.0)
Chronic kidney disease	6454 (5.6)	812 (4.7)
Chronic lung disease (not asthma or pulmonary fibrosis)	9448 (8.2)	1048 (6.1)
Chronic neurologic disorder (not dementia)	11 194 (9.7)	1119 (6.5)
Congestive heart failure	6148 (5.3)	629 (3.7)
Coronary artery disease	10 878 (9.4)	1235 (7.2)
Dementia	4869 (4.2)	720 (4.2)
Diabetes	16 699 (14.5)	2901 (16.9)
Dialysis	690 (0.6)	75 (0.4)
Dyslipidemia	20 408 (17.7)	2657 (15.5)
Hypertension	37 2350 (32.2)	5116 (29.8)
Hypothyroidism	9390 (8.1)	1234 (7.2)
Mild liver disease	1375 (1.2)	119 (0.7)
Moderate or severe liver disease	1047 (0.9)	96 (0.6)
Obesity (clinical impression)	2010 (1.7)	326 (1.9)
Organ transplant	603 (0.5)	111 (0.7)
Rheumatologic disorder	10 814 (9.4)	939 (5.5)
Past malignant neoplasm	5807 (5.0)	568 (3.3)
Psychiatric condition	21 882 (18.9)	1764 (10.3)
Pulmonary fibrosis	326 (0.3)	41 (0.2)
Symptoms reported		
Cough	24 801 (21.5)	9325 (54.3)
Fever	19 792 (17.1)	7544 (43.9)
Chills	9527 (8.2)	3450 (20.1)
Shortness of breath	33 552 (29.0)	7126 (41.5)
Muscle aches (myalgia)	6274 (5.4)	3107 (18.1)
Fatigue or malaise	23 019 (19.9)	5721 (33.3)
Diarrhea	11 205 (9.7)	2501 (14.6)
Headache	11 958 (10.4)	3388 (19.7)
Dysgeusia or anosmia	621 (0.5)	1019 (5.9)
Nausea or vomiting	24 963 (21.6)	3536 (20.6)
Current tobacco use^a^	12 592 (10.9)	888 (5.2)
Current illicit substance use^a^	10 323 (8.9)	807 (4.7)
Oxygen required in ED^a^	10 242 (8.9)	2892 (16.8)
Severe COVID-19^b^	NA	4130 (24.0)
Hospital admission	56 068 (48.5)	6669 (38.8)
Mechanical ventilation	2378 (2.1)	690 (4.0)
In-hospital death	4056 (3.5)	1154 (6.7)

Abbreviations: ED, emergency department; NA, not applicable.

^a^ Missing or unknown data for the following variables are body temperature: 4319 of 115 586 (3.7%) for COVID-19–negative patients, 598 of 17 174 (3.5%) for COVID-19–positive patients; heart rate: 1765 of 115 586 (1.5%) for COVID-19–negative patients, 249 of 17 174 (1.4%) for COVID-19–positive patients; oxygen saturation: 2151 of 115 586 (1.9%) for COVID-19–negative patients, 267 of 17 174 (1.6%) for COVID-19–positive patients; respiratory rate: 3149 of 115 586 (2.7%) for COVID-19–negative patients, 636 of 17 174 (3.7%) for COVID-19–positive patients; systolic blood pressure: 4526 of 115 586 (3.9%) for COVID-19–negative patients, 833 of 17 174 (4.9%) for COVID-19–positive patients; current tobacco use: 73 887 of 115 586 (63.9%) for COVID-19–negative patients, 12 358 of 17 174 (71.2%) for COVID-19–positive patients; current illicit substance use: 79 142 of 115 586 (68.4%) for COVID-19–negative patients, 13 151 of 17 174 (76.6%) for COVID-19–positive patients; and oxygen required in the ED: 6361 of 115 586 (5.5%) for COVID-19–negative patients, 283 of 17 174 (1.6%) for COVID-19–positive patients.

^b^ Criteria for severe COVID-19 were met if the patient had an oxygen saturation of less than 90% while breathing room air, a respiratory rate of more than 30 breaths/min, or signs of severe respiratory distress as documented in the ED record.

Of the 17 174 SARS-CoV-2–positive patients, 538 (3.1%) had an initially negative test result. Of the 538 patients with an initial negative test result, 434 (80.7%) tested positive within 14 days and were thus considered to have had at least 1 false-negative test result. All other patients tested positive after the follow-up period and were not considered to have had an initial false-negative test result. Among patients with false-negative test results, the mean (SD) number of days between their first negative test result and the subsequent positive test result was 5.5 (4.1) days.

**Figure 2.**
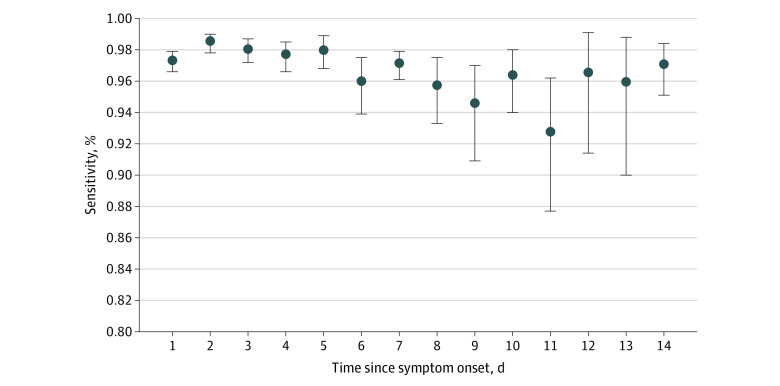
SARS-CoV-2 Nucleic Acid Amplification Test (NAAT) Sensitivity by Symptom Duration Sensitivity for the first SARS-CoV-2 NAAT performed in the hospital for 96 232 patients who reported a date of symptom onset. Vertical lines indicate 95% CIs.

In our cohort, the sensitivity of the SARS-CoV-2 NAAT was 96.2% (17 070 of 17 740 [95% CI, 95.9%-96.4%]) among all of the tests performed. The sensitivity of the SARS-CoV-2 NAAT by symptom duration ranged from a high of 97.7% (1710 of 1751) (95% CI, 96.8%-98.3%) on day 2 of symptoms to a low of 90.4% (170 of 188) (95% CI, 85.3%-94.2%) on day 11 of symptoms (Figure 2; eTable 4 in Supplement 1). The sensitivity of the SARS-CoV-2 NAAT among patients who reported COVID-19 symptoms was 97.1% (11 870 of 12 225 [95% CI, 96.7%-97.3%]) compared with 87.6% (812 of 927 [95% CI, 85.2%-89.6%]) among patients without COVID-19 symptoms. Among patients who identified as health care workers or who lived in long-term care facilities, the sensitivity was 94.2% (937 of 995 [95% CI, 92.4%-95.5%]) compared with 96.6% (11 745 of 12 157 [95% CI, 96.2%-96.8%]) among non–health care workers or those living at home. Most NAATs were performed using nasopharyngeal swabs (eTable 3 in Supplement 1).

**Figure 3.**
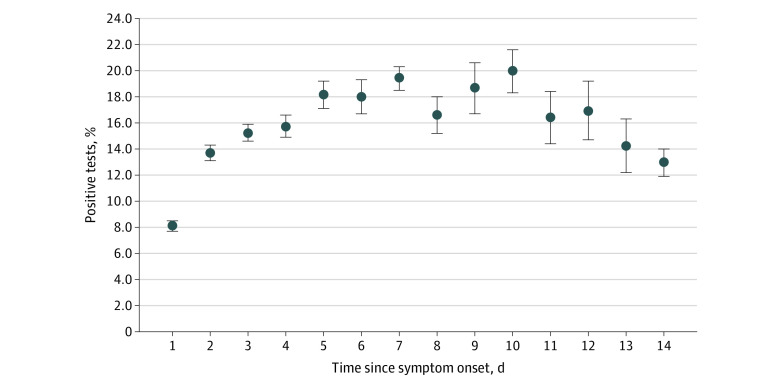
SARS-CoV-2 Nucleic Acid Amplification Test (NAAT) Diagnostic Yield by Symptom Duration Diagnostic yield at the time of the first SARS-CoV-2 NAAT among 96 232 patients who reported a date of symptom onset. Vertical lines indicate 95% CIs.

In our cohort, the diagnostic yield of the NAAT was 12.0% (18 985 of 158 004 [95% CI, 11.8%-12.2%]). Among patients who reported a date of symptom onset, the diagnostic yield was 12.2% (13 454 of 110 453 [95% CI, 11.9%-12.3%]). The lowest diagnostic yield was 8.1% (1686 of 20 719 [95% CI, 7.7%-8.5%]) among patients presenting within the first 24 hours of symptom onset, and the highest diagnostic yield was 20.0% (445 of 2229 [95% CI, 18.3%-21.6%]) on day 10 after symptom onset (Figure 3; eTable 5 in Supplement 1).

When examining the association between patient factors and false-negative test results, we found that patients who were older (adjusted odds ratio [aOR], 1.02 [95% CI, 1.01-1.03]), with chronic kidney disease (aOR, 1.96 [95% CI, 1.35-2.84]) or psychiatric conditions (aOR, 4.85 [95% CI, 1.36-2.52]), and those reporting abdominal pain (aOR, 1.73 [95% CI, 1.27-2.34]) were more likely to have an initial false-negative test result (Table 2). Patients experiencing symptoms typically associated with COVID-19, including cough (aOR, 0.46 [95% CI, 0.35-0.60]), fever (aOR, 0.66 [95% CI, 0.50-0.86]), and muscle aches (aOR, 0.43 [95% CI, 0.27-0.68]), were less likely to have a false-negative test result.

**Table 2.** Crude and Adjusted Analyses of Factors Associated With an Initial False-Negative SARS-CoV-2 NAAT Result

Variable	Odds ratio (95% CI)		*P* value
	Unadjusted	Adjusted	
Female	0.79 (0.59-1.05)	0.85 (0.67-1.07)	.17
Age	1.02 (1.02-1.03)	1.02 (1.01-1.03)	<.001
COVID-19 incidence, cases per 100 000^a^			
<3	1 [Reference]	1 [Reference]	.60
3-15	1.98 (1.01-3.39)	1.26 (0.79-2.00)	
16-37	1.94 (0.99-3.79)	1.08 (0.68-1.72)	
>37	2.02 (0.96-4.28)	1.22 (0.70-2.14)	
Time between symptom onset and first swab, d			
<24 h	1 [Reference]	1 [Reference]	.56
1-3	0.63 (0.43-0.95)	0.85 (0.62-1.17)	
4-10	0.75 (0.54-1.05)	0.82 (0.61-1.09)	
11-14	0.85 (0.48-1.49)	0.86 (0.52-1.42)	
Common comorbid conditions			
Active malignant neoplasm	1.85 (1.07-3.20)	NA	NA
Asthma	0.58 (0.29-1.14)	NA	NA
Atrial fibrillation	2.73 (1.73-4.29)	NA	NA
Chronic kidney disease	4.10 (2.71-6.20)	1.96 (1.35-2.84)	<.001
Chronic lung disease	2.05 (1.31-3.21)	NA	NA
Chronic neurologic disorder	1.96 (1.25-3.06)	NA	NA
Congestive heart failure	1.70 (0.94-3.07)	NA	NA
Coronary artery disease	1.54 (0.99-2.40)	NA	NA
Dementia	3.18 (1.91-5.29)	NA	NA
Diabetes	1.59 (1.15-2.20)	NA	NA
Dyslipidemia	2.25 (1.65-3.07)	NA	NA
Hypertension	2.37 (1.78-3.12)	NA	NA
Hypothyroidism	1.30 (0.82-2.09)	NA	NA
Past malignant neoplasm	2.28 (1.34-3.88)	NA	NA
Psychiatric condition	2.00 (1.37-2.92)	4.85 (1.36-2.52)	<.001
Rheumatologic disorder	1.86 (1.17-2.98)	NA	NA
Symptoms reported			
Abdominal pain	2.12 (1.51-2.98)	1.73 (1.27-2.34)	<.001
Chest pain	0.85 (0.62-1.17)	NA	NA
Chills	0.84 (0.58-1.21)	NA	NA
Confusion	2.68 (1.86-3.87)	NA	NA
Cough	0.44 (0.33-0.58)	0.46 (0.35-0.60)	<.001
Diarrhea	0.92 (0.56-1.51)	NA	NA
Dizziness	0.96 (0.59-1.56)	NA	NA
Dysgeusia	0.53 (0.27-1.05)	NA	NA
Fatigue	0.92 (0.69-1.23)	NA	NA
Fever	0.52 (0.39-0.69)	0.66 (0.50-0.86)	.002
Headache	0.54 (0.37-0.80)	NA	NA
Muscle ache	0.27 (0.16-0.45)	0.43 (0.27-0.68)	<.001
Nausea or vomiting	0.96 (0.69-1.34)	NA	NA
Respiratory distress	1.71 (1.16-2.53)	NA	NA
Sputum production	0.71 (0.41-1.22)	NA	NA
Sore throat	0.29 (0.16-0.52)	NA	NA
Shortness of breath	1.13 (0.85-1.49)	NA	NA

Abbreviations: NA, not applicable; NAAT, nucleic acid amplification test.

^a^ The 7-day regional COVID-19 incidence refers to the moving mean incident COVID-19 case count of the patients’ health region at the time of their emergency department visit.

## Discussion

Our aim was to evaluate the clinical sensitivity of SARS-CoV-2 NAATs in a nationally representative sample of consecutive patients presenting to the hospital and to assess the association of symptom onset with diagnostic accuracy. Our results indicate high sensitivity of the test, with little variation during the first 14 days of symptoms. The diagnostic yield, however, varied by symptom duration, peaking for patients presenting at around day 10 of symptoms, suggesting that those who present to the emergency department with prolonged respiratory symptoms are more likely to receive a diagnosis of COVID-19. Our results did not vary significantly for health care workers or residents of long-term care facilities, who may be tested more frequently. We found that patients presenting with typical COVID-19 symptoms had lower odds of a false-negative test result compared with other patients, while those reporting comorbid conditions, such as mental health illness—which may have been associated with psychiatric admissions and lower pretest probability of a COVID-19 diagnosis—had higher odds of a false-negative test result. This finding is consistent with the spectrum bias or spectrum effect that is commonly observed when diagnostic tests are evaluated in populations with different clinical pretest probabilities of disease.^21^

Reviews have summarized the diagnostic test performance of the NAAT in cohorts of mostly hospitalized patients recruited in China during the early pandemic when case definitions were evolving.^19,22^ Most studies used repeated testing with the NAAT as the criterion standard, either with the same assay or a different assay, and reported pooled clinical sensitivities between 57.9% and 94.2%. Included studies were at high risk of bias, with most not describing consecutive cohorts, and many enriched for samples with positive test results, making the pooled results susceptible to spectrum bias. A study completed in the United States estimated the clinical sensitivity of a single SARS-CoV-2 NAAT in 34 348 hospitalized patients with documentation of at least 2 symptoms attributable to COVID-19 who were retested within 48 hours during the early pandemic.^23^ The estimated sensitivity of a single NAAT was between 82% and 97% among this high-risk group. A Canadian study reported on initially negative SARS-CoV-2 NAAT results that were followed by a positive test result within 14 days using Public Health Laboratory samples.^24^ They estimated the sensitivity of the initial NAAT at 90.7%, which they attributed to low SARS-CoV-2 concentrations detectable by the initial test. Our study compared favorably with these studies. We were able to capture a large consecutive sample of patients presenting to 47 sites across Canada, reflecting hospital practice. We had strict measures in place to ensure a consecutive sample, minimizing spectrum bias in our study. Our results indicate high sensitivity for infections prompting emergency department visits and hospitalizations, indicating little value for repeated testing unless the clinical suspicion for disease remains very high.

Reviews have pooled data from early studies to assess the association of symptom duration with diagnostic test performance and have estimated the highest test sensitivity at 89% between zero and 4 days after symptom onset, with decreasing sensitivity of 81% by the time of hospital admission.^3,4,7^ Included studies were at substantial risk of bias for participant selection owing to nonconsecutive enrollment of hospitalized patients. The applicability of these results to the global context is questionable because cohort studies from North America indicate that patients typically present to the hospital 5 days into their symptomatic illness.^25^ Similarly, a single-center retrospective study from early 2020 that enrolled mainly hospitalized patients with high rates of test positivity estimated sensitivity at more than 90% during the first 5 days after symptom onset, 76% to 84% between 6 and 8 days, and 70% to 71% between 9 to 11 days, decreasing to 30% at day 21.^5^ In our study, the clinical diagnostic test characteristics did not vary substantially by symptom duration, and sensitivity was preserved within 14 days from symptom onset, albeit with wider 95% CIs given the smaller sample sizes of patients being tested later.

One review compared the diagnostic yields of different specimens and reported lower yields with nasal, oropharyngeal, and saliva swab specimens compared with nasopharyngeal swab specimens.^26^ Another study of asymptomatic or mildly symptomatic patients reported similar pooled sensitivities for saliva speciments at 83.2% and nasopharyngeal swab speciments at 84.8%.^27^ We were unable to complete evaluations by specimen type because almost all of the specimens were nasopharyngeal. The diagnostic yield of testing in our study varied, which is likely a reflection of the difference in patients who present over time, with patients presenting after 1 week of unresolving symptoms being more likely to have COVID-19 and more likely to have severe disease.

### Strengths and Limitations

This study has some strengths, including enrollment of a consecutive sample of patients presenting to the emergency department that included those being admitted to the hospital and frequently undergoing repeated testing.^27^ By using a uniform sample of patients tested in emergency departments, we controlled for health access variables, which would be difficult to control for in data sets containing the test results of both community- and hospital-based test sites. We used a composite reference standard that included all of the tests a patient had undergone, reporting the diagnostic test characteristics of the first test recorded, similar to prior studies but using a much larger sample size. We were able to perform sensitivity analyses, excluding asymptomatic patients and excluding patients who may be tested at regular intervals for screening purposes owing to their work or living situation.^22^ We were able to control for 7-day COVID-19 incident cases in the health region of the patient’s postal code of residence, thus controlling for baseline disease prevalence at the time of the patient’s presentation, which can be associated with clinical diagnostic test performance.^28^

Our study also has some limitations. Our data were collected retrospectively; thus, information about specimen quality was not captured. Similar to prior studies, we were unable to control for new COVID-19 exposures over time. Like prior studies that used consecutive tests on the same patients, we may have overestimated the rate of false-negative test results (and underestimated the sensitivity of the test) because patients could have been newly exposed to COVID-19 during follow-up. As a result, we may have underestimated sensitivity. We chose to exclude sites that were unable to enroll 99% or more of eligible patients to minimize selection bias. Although this decision could have led to bias at the site level, whereby sites most overwhelmed during the pandemic and unable to follow study procedures may have been least likely to participate, 47 of 50 CCEDRRN sites met this requirement, indicating that the risk of site-level selection bias was low. Although we included all repeated testing performed during an emergency department revisit or at readmission, we were unable to include the results of community-based NAATs completed after hospital discharge, which may have led us to underestimate false-negative test results and overestimate sensitivity. However, these infections would have been mild and of less clinical concern because the patients did not require subsequent use of acute care. Finally, owing to the observational nature of this study, we were limited in ascertaining a truly independent criterion standard, such as repeated testing for the same patient by a different laboratory. Repeated testing was initiated at the discretion of treating physicians and thus likely reflected those patients for whom the physicians had a high index of suspicion of disease. Despite these limitations, we believe our study provides a robust clinical estimation of NAAT sensitivity and can guide emergency department physicians and admitting teams in their interpretation of diagnostic test results at the time patients present.

## Conclusions

The high diagnostic sensitivity of the NAAT suggests that 1 negative test result can effectively rule out SARS-CoV-2 infection for most patients presenting to the emergency department, including those being tested at admission. Only patients with a very high clinical pretest probability and an initially negative test result warrant repeated testing.

## Notes

Supplement 1. **eTable 1*.*** Patient Enrollment Across Participating Sites **eTable 2*.*** Patient Characteristics by Symptom Duration at the Time of Their First SARS-CoV-2 NAAT (1 to 7 Days) **eTable 3*.*** Patient Characteristics by Symptom Duration at the Time of Their First SARS-CoV-2 NAAT (8 to 14 Days) **eFigure*.*** Treatments and Outcomes Among Patients by Symptom Duration at the Time of Their First SARS-CoV-2 NAAT Among 96,232 Patients Who Reported a Date of Symptom Onset **eTable 4.** Sensitivity of SARS-CoV-2 NAATs, by Symptom Duration Among 12,726 SARS-CoV-2 Positive Patients Who Reported a Date of Symptom Onset **eTable 5.** Diagnostic Yield by Symptom Duration at the Time of the First SARS-CoV-2 NAAT Among 96,232 Patients Who Reported a Date of Symptom Onset

Supplement 2. **Nonauthor Collaborators.** The Canadian COVID-19 Emergency Department Rapid Response Network (CCEDRRN) investigators for the Network of Canadian Emergency Researchers and the Canadian Critical Care Trials Group

## References

[1] Dinnes J, Deeks JJ, Berhane S, ; Cochrane COVID-19 Diagnostic Test Accuracy Group. Rapid, point-of-care antigen and molecular-based tests for diagnosis of SARS-CoV-2 infection. Cochrane Database Syst Rev. 2021;3:CD013705. doi:10.1002/14651858.CD013705 33760236 PMC8078597

[2] Infectious Diseases Society of America. IDSA guidelines on the diagnosis of COVID-19: molecular diagnostic testing. Updated December 23, 2020. Accessed March 10, 2022. https://www.idsociety.org/practice-guideline/covid-19-guideline-diagnostics/#

[3] Kucirka LM, Lauer SA, Laeyendecker O, Boon D, Lessler J. Variation in false-negative rate of reverse transcriptase polymerase chain reaction-based SARS-CoV-2 tests by time since exposure. Ann Intern Med. 2020;173(4):262-267. doi:10.7326/M20-1495 32422057 PMC7240870

[4] Mallett S, Allen AJ, Graziadio S, . At what times during infection is SARS-CoV-2 detectable and no longer detectable using RT-PCR–based tests? a systematic review of individual participant data. BMC Med. 2020;18(1):346. doi:10.1186/s12916-020-01810-8 33143712 PMC7609379

[5] Miller TE, Garcia Beltran WF, Bard AZ, . Clinical sensitivity and interpretation of PCR and serological COVID-19 diagnostics for patients presenting to the hospital. FASEB J. 2020;34(10):13877-13884. doi:10.1096/fj.202001700RR 32856766 PMC7461169

[6] Dugdale CM, Anahtar MN, Chiosi JJ, . Clinical, laboratory, and radiologic characteristics of patients with initial false-negative severe acute respiratory syndrome coronavirus 2 nucleic acid amplification test results. Open Forum Infect Dis. 2020;8(1):ofaa559. doi:10.1093/ofid/ofaa559 34164560 PMC7717411

[7] Wikramaratna PS, Paton RS, Ghafari M, Lourenço J. Estimating the false-negative test probability of SARS-CoV-2 by RT-PCR. Euro Surveill. 2020;25(50):2000568. doi:10.2807/1560-7917.ES.2020.25.50.2000568 33334398 PMC7812420

[8] Skittrall JP, Wilson M, Smielewska AA, . Specificity and positive predictive value of SARS-CoV-2 nucleic acid amplification testing in a low-prevalence setting. Clin Microbiol Infect. 2021;27(3):469.e9-469.e15. doi:10.1016/j.cmi.2020.10.003 33068757 PMC7554481

[9] Jayk Bernal A, Gomes da Silva MM, Musungaie DB, ; MOVe-OUT Study Group. Molnupiravir for oral treatment of COVID-19 in nonhospitalized patients. N Engl J Med. 2022;386(6):509-520. doi:10.1056/NEJMoa2116044 34914868 PMC8693688

[10] Hammond J, Leister-Tebbe H, Gardner A, . Oral nirmatrelvir for high-risk, nonhospitalized adults with COVID-19. N Engl J Med. 2022;386(15):1397-1408. doi:10.1056/NEJMoa2118542 35172054 PMC8908851

[11] Hohl CM, Rosychuk RJ, McRae AD, ; Canadian COVID-19 Emergency Department Rapid Response Network investigators; Network of Canadian Emergency Researchers; Canadian Critical Care Trials Group. Development of the Canadian COVID-19 Emergency Department Rapid Response Network population-based registry: a methodology study. CMAJ Open. 2022;9(1):E261-E270. doi:10.9778/cmajo.20200290 33731427 PMC8096396

[12] Hohl CM, Rosychuk RJ, Hau JP, ; Canadian COVID-19 Rapid Response Network (CCEDRRN) investigators for the Network of Canadian Emergency Researchers, for the Canadian Critical Care Trials Group. Treatments, resource utilization, and outcomes of COVID-19 patients presenting to emergency departments across pandemic waves: an observational study by the Canadian COVID-19 Emergency Department Rapid Response Network (CCEDRRN). CJEM. 2022;24(4):397-407. doi:10.1007/s43678-022-00275-3 35362857 PMC8972682

[13] McRae AD, Hohl CM, Rosychuk R, ; Canadian COVID-19 Emergency Department Rapid Response Network (CCEDRRN) investigators for the Network of Canadian Emergency Researchers and the Canadian Critical Care Trials Group. CCEDRRN COVID-19 Infection Score (CCIS): development and validation in a Canadian cohort of a clinical risk score to predict SARS-CoV-2 infection in patients presenting to the emergency department with suspected COVID-19. BMJ Open. 2021;11(12):e055832. doi:10.1136/bmjopen-2021-055832 34857584 PMC8640195

[14] Hohl CM, Rosychuk RJ, Archambault PM, ; Canadian COVID-19 Emergency Department Rapid Response Network (CCEDRRN) investigators for the Network of Canadian Emergency Researchers and the Canadian Critical Care Trials Group. The CCEDRRN COVID-19 Mortality Score to predict death among nonpalliative patients with COVID-19 presenting to emergency departments: a derivation and validation study. CMAJ Open. 2022;10(1):E90-E99. doi:10.9778/cmajo.20210243 35135824 PMC9259439

[15] Bossuyt PM, Reitsma JB, Bruns DE, ; STARD Group. STARD 2015: an updated list of essential items for reporting diagnostic accuracy studies. BMJ. 2015;351:h5527. doi:10.1136/bmj.h5527 26511519 PMC4623764

[16] ISARIC. Clinical data collection—the COVID-19 case report forms (CRFs). 2020. Accessed March 9, 2021. https://isaric.org/research/covid-19-clinical-research-resources/covid-19-crf/

[17] Green DA, Zucker J, Westblade LF, . Clinical performance of SARS-CoV-2 molecular tests. J Clin Microbiol. 2020;58(8):e00995-20. doi:10.1128/JCM.00995-20 32513858 PMC7383556

[18] Chandler CM, Bourassa L, Mathias PC, Greninger AL. Estimating the false-positive rate of highly automated SARS-CoV-2 nucleic acid amplification testing. J Clin Microbiol. 2021;59(8):e0108021. doi:10.1128/JCM.01080-21 33972455 PMC8288255

[19] Mustafa Hellou M, Górska A, Mazzaferri F, . Nucleic acid amplification tests on respiratory samples for the diagnosis of coronavirus infections: a systematic review and meta-analysis. Clin Microbiol Infect. 2021;27(3):341-351. doi:10.1016/j.cmi.2020.11.002 33188933 PMC7657614

[20] World Health Organization. Living guidance for clinical management of COVID-19. 2021. Accessed March 9, 2021. https://www.who.int/publications/i/item/WHO-2019-nCoV-clinical-2021-2

[21] Willis BH. Spectrum bias—why clinicians need to be cautious when applying diagnostic test studies. Fam Pract. 2008;25(5):390-396. doi:10.1093/fampra/cmn051 18765409

[22] Axell-House DB, Lavingia R, Rafferty M, Clark E, Amirian ES, Chiao EY. The estimation of diagnostic accuracy of tests for COVID-19: a scoping review. J Infect. 2020;81(5):681-697. doi:10.1016/j.jinf.2020.08.043 32882315 PMC7457918

[23] Ridgway JP, Pisano J, Landon E, . Clinical sensitivity of SARS-CoV-2 nucleic acid amplification tests for diagnosing COVID-19. Open Forum Infect Dis. 2020;7(8):ofaa315. doi:10.1093/ofid/ofaa315 32818146 PMC7423294

[24] Kanji JN, Zelyas N, MacDonald C, . False negative rate of COVID-19 PCR testing: a discordant testing analysis. Virol J. 2021;18(1):13. doi:10.1186/s12985-021-01489-0 33422083 PMC7794619

[25] Hohl CM, Rosychuk RJ, Hau JP, . Treatments, resource utilization, and outcomes of COVID-19 patients presenting to emergency departments across pandemic waves: an observational study by the Canadian COVID-19 Emergency Department Rapid Response Network (CCEDRRN). *medRxiv*. Preprint posted online August 1, 2021. doi:10.1101/2021.07.30.21261288 PMC8972682 35362857

[26] Lee RA, Herigon JC, Benedetti A, Pollock NR, Denkinger CM. Performance of saliva, oropharyngeal swabs, and nasal swabs for SARS-CoV-2 molecular detection: a systematic review and meta-analysis. J Clin Microbiol. 2021;59(5):e02881-20. doi:10.1128/JCM.02881-20 33504593 PMC8091856

[27] Butler-Laporte G, Lawandi A, Schiller I, . Comparison of saliva and nasopharyngeal swab nucleic acid amplification testing for detection of SARS-CoV-2: a systematic review and meta-analysis. JAMA Intern Med. 2021;181(3):353-360. doi:10.1001/jamainternmed.2020.8876 33449069 PMC7811189

[28] Braunstein GD, Schwartz L, Hymel P, Fielding J. False positive results with SARS-CoV-2 RT-PCR tests and how to evaluate a RT-PCR–positive test for the possibility of a false positive result. J Occup Environ Med. 2021;63(3):e159-e162. doi:10.1097/JOM.0000000000002138 33405498 PMC7934325

