# Supplementary material for: Sensitivity and Diagnostic Yield of the First SARS-CoV-2 Nucleic Acid Amplification Test Performed for Patients Presenting to the Hospital
Source: JAMA Netw Open. 2022 Oct 12;5(10):e2236288. doi: 10.1001/jamanetworkopen.2022.36288 (PMC9557877; doi:10.1001/jamanetworkopen.2022.36288)
Supplement: Supplement 1. — eTable 1. Patient Enrollment Across Participating Sites eTable 2. Patient Characteristics by Symptom Duration at the Time of Their First SARS-CoV-2 NAAT (1 to 7 Days) eTable 3. Patient Characteristics by Symptom Duration at the Time of Their First SARS-CoV-2 NAAT (8 to 14 Days) eFigure. Treatments and Outcomes Among Patients by Symptom Duration at the Time of Their First SARS-CoV-2 NAAT Among 96,232 Patients Who Reported a Date of Symptom Onset eTable 4. Sensitivity of SARS-CoV-2 NAATs, by Symptom Duration Among 12,726 SARS-CoV-2 Positive Patients Who Reported a Date of Symptom Onset eTable 5. Diagnostic Yield by Symptom Duration at the Time of the First SARS-CoV-2 NAAT Among 96,232 Patients Who Reported a Date of Symptom Onset [file jamanetwopen-e2236288-s001.pdf]

Supplementary Online Content

Hohl CM, Hau JP, Vaillancourt S, et al; CCEDRRN investigators for the Network of Canadian Emergency Researchers and the Canadian Critical Care Trials Group. Sensitivity and diagnostic yield of the first SARS-CoV-2 nucleic acid amplification test performed for patients presenting to the hospital. *JAMA Netw Open.* 2022;5(10):e2236288. doi:10.1001/jamanetworkopen.2022.36288

eTable 1. Patient Enrollment Across Participating Sites
eTable 2. Patient Characteristics by Symptom Duration at the Time of Their First SARS-CoV-2 NAAT (1 to 7 Days)
eTable 3. Patient Characteristics by Symptom Duration at the Time of Their First SARS-CoV-2 NAAT (8 to 14 Days)
eFigure. Treatments and Outcomes Among Patients by Symptom Duration at the Time of Their First SARS-CoV-2 NAAT Among 96,232 Patients Who Reported a Date of Symptom Onset
eTable 4. Sensitivity of SARS-CoV-2 NAATs, by Symptom Duration Among 12,726 SARS-CoV-2 Positive Patients Who Reported a Date of Symptom Onset
eTable 5. Diagnostic Yield by Symptom Duration at the Time of the First SARS-CoV-2 NAAT Among 96,232 Patients Who Reported a Date of Symptom Onset

This supplementary material has been provided by the authors to give readers additional information about their work.

© 2022 Hohl CM et al. *JAMA Network Open.*

eTable 1. Patient Enrollment Across Participating Sites

Site Name	Province	Start Date	End Date	Number of patients
Abbottsford Regional Hospital	British Columbia	2020-03-20	2021-05-31	2,294
Eagle Ridge Hospital	British Columbia	2020-03-01	2021-05-30	902
Kelowna General Hospital	British Columbia	2020-03-20	2021-05-31	560
Lions Gate Hospital	British Columbia	2020-03-01	2021-05-31	1,288
Mount Saint Joseph's Hospital	British Columbia	2020-03-01	2021-12-31	725
Royal Columbian Hospital	British Columbia	2020-03-01	2021-05-31	1,974
Royal Inland Hospital	British Columbia	2020-03-16	2021-05-30	272
Saint Paul's Hospital	British Columbia	2020-03-01	2021-09-30	10,216
Surrey Memorial Hospital	British Columbia	2020-03-19	2021-12-03	6,030
Vancouver General Hospital	British Columbia	2020-03-01	2021-08-31	12,531
Foothills Medical Centre	Alberta	2020-03-01	2021-11-30	2,528
Northeast Community Health Centre	Alberta	2020-03-23	2021-01-29	407
Peter Lougheed Centre	Alberta	2020-03-01	2021-11-30	14,604
Rockyview General Hospital	Alberta	2020-03-01	2021-11-30	2,291
Royal Alexandra Hospital	Alberta	2020-03-16	2020-12-31	840
South Health Campus Medical Centre	Alberta	2020-03-01	2021-11-30	11,561
University of Alberta Hospital	Alberta	2020-03-23	2021-01-26	73
Royal University Hospital	Saskatchewan	2020-03-17	2021-12-31	7,473
Saskatoon City Hospital	Saskatchewan	2020-03-01	2021-02-25	184
St Paul’s Hospital	Saskatchewan	2020-03-06	2021-12-27	686
Hamilton General Hospital	Ontario	2020-03-20	2020-12-30	204
Health Science North Hospital	Ontario	2020-05-14	2021-07-31	8124
Hotel Dieu Hospital	Ontario	2020-03-14	2021-11-30	290
Juravinski Hospital	Ontario	2020-03-09	2020-12-31	249
Kingston General Hospital	Ontario	2020-03-12	2021-11-30	1,619
London Health Sciences Center	Ontario	2020-03-01	2021-05-31	638
North York General Hospital	Ontario	2020-05-14	2020-06-04	1,006
Sunnybrook Health Sciences Centre	Ontario	2020-05-14	2021-12-26	13,042

© 2022 Hohl CM et al. *JAMA Network Open.*

The Ottawa Hospital - Civic Campus	Ontario	2020-03-11	2021-05-27	985
The Ottawa Hospital - General Campus	Ontario	2020-03-05	2021-05-29	682
Toronto Western Hospital	Ontario	2020-09-01	2021-07-28	9,259
Centre Hospitalier de l'Université Laval	Quebec	2020-03-12	2021-01-18	189
Hôpital de l'Enfant-Jésus	Quebec	2020-05-04	2021-06-30	11,603
Hôpital du Sacré-Coeur	Quebec	2020-03-18	2021-11-04	2,062
Hôpital du Saint-Sacrement	Quebec	2020-03-10	2021-01-17	115
Hôpital Saint-François d'Assise	Quebec	2020-03-12	2021-01-18	307
Hôtel-Dieu de Lévis	Quebec	2020-03-10	2021-12-31	1,102
Hôtel-Dieu de Québec	Quebec	2020-03-28	2021-01-16	74
Institut universitaire de cardiologie et de pneumologie de Québec	Quebec	2020-03-23	2021-01-14	540
Jewish General Hospital	Quebec	2020-03-01	2021-09-30	19,036
L'hôpital Royal Victoria	Quebec	2020-03-12	2021-11-30	1,458
Montréal General Hospital	Quebec	2020-03-17	2021-11-24	562
Saint John Regional Hospital	New Brunswick	2020-03-12	2021-10-25	314
Cobequid Community Health Centre	Nova Scotia	2020-03-01	2021-12-31	214
Dartmouth General Hospital	Nova Scotia	2020-03-01	2021-12-31	337
Halifax Infirmary	Nova Scotia	2020-03-01	2021-11-24	476
Hants Community Hospital	Nova Scotia	2020-03-01	2021-12-28	129

© 2022 Hohl CM et al. *JAMA Network Open.*

eTable 2. Patient Characteristics by Symptom Duration at the Time of Their First SARS-CoV-2 NAAT (1 to 7 Days)

Variables	Number of days between symptoms onset and first hospital COVID test						
	1 Day (n=3,341) Patients, No. (%)	2 Days (n=1,730) Patients, No. (%)	3 Days (n=1,745) Patients, No. (%)	4 Days (n=1,129) Patients, No. (%)	5 Days (n=902) Patients, No. (%)	6 Days (n=533) Patients, No. (%)	7 Days (n=1,506) Patients, No. (%)
**Swab Method**							
Nasopharyngeal Swab	2,868 (85.8)	1,572 (90.5)	1,605 (91.9)	1,030 (91.2)	816 (90.5)	474 (88.9)	1,350 (89.6)
Mouth / Saliva	29 (0.8)	11 (0.6)	12 (0.7)	11 (0.9)	< 5	< 5	19 (1.3)
Mid-Turbinate Swab	153 (4.6)	44 (2.5)	42 (2.4)	28 (2.5)	26 (2.9)	22 (4.1)	34 (2.3)
Saline / Water Gargle	27 (0.8)	17 (1.0)	16 (0.9)	9 (0.8)	8 (0.9)	< 5	6 (0.4)
Sputum	-	< 5	< 5	-	-	< 5	< 5
Brochalveolar Lavage	-	< 5	-	-	-	-	-
Trachial Aspirate	16 (0.5)	< 5	-	< 5	< 5	< 5	< 5
Blood test	< 5	-	-	< 5	-	-	-
Nasal wash	-	< 5	-	< 5	-	-	< 5
Other	243 (7.3)	79 (4.6)	66 (3.8)	46 (4.1)	44 (4.8)	28 (5.3)	86 (5.7)
Missing	< 5	< 5	< 5	< 5	< 5	-	< 5
**Admitted**	1,288 (38.5)	507 (29.3)	558 (32.0)	392 (34.7)	297 (32.9)	180 (33.7)	577 (38.3)
**ICU Admission**	215 (6.4)	87 (5.0)	140 (8.0)	82 (7.3)	67 (7.4)	52 (9.7)	173 (11.5)
**Severe COVID-19**	615 (18.4)	342 (19.8)	396 (22.7)	273 (24.1)	240 (26.6)	158 (29.6)	427 (28.4)
**Intubated**	130 (3.9)	58 (3.4)	81 (4.6)	42 (3.7)	33 (3.7)	29 (5.4)	92 (6.1)
**Steroids**	620 (18.6)	333 (19.3)	387 (22.2)	269 (23.8)	199 (22.1)	138 (25.9)	428 (28.4)
**Death**	246 (7.4)	106 (6.1)	116 (6.6)	61 (5.4)	38 (4.2)	22 (4.1)	91 (6.0)

© 2022 Hohl CM et al. *JAMA Network Open.*

eTable 3. Patient Characteristics by Symptom Duration at the Time of Their First SARS-CoV-2 NAAT (8 to 14 Days)

	Number of days between symptoms onset and first hospital COVID test						
**Variables**	**8 Days (n=408) Patients, No. (%)**	**9 Days (n=222) Patients, No. (%)**	**10 Days (n=385) Patients, No. (%)**	**11 Days (n=156) Patients, No. (%)**	**12 Days (n=93) Patients, No. (%)**	**13 Days (n=93) Patients, No. (%)**	**14 Days (n=478) Patients, No. (%)**
**Swab Method**							
Nasopharyngeal Swab	364 (89.2)	201 (90.5)	174 (45.2)	141 (90.3)	93 (94.9)	82 (88.2)	431 (90.2)
Mouth / Saliva	< 5	< 5	< 5	< 5	-	-	< 5
Mid-Turbinate Swab	18 (4.4)	8 (3.6)	16 (4.2)	5 (3.2)	< 5	< 5	18 (3.6)
Saline / Water Gargle	< 5	< 5	< 5	< 5	-	-	< 5
Sputum	-	-	-	-	-	-	-
Brochalveolar Lavage	-	-	< 5	< 5	-	-	-
Trachial Aspirate	-	< 5	< 5	< 5	-	< 5	< 5
Blood test	-	-	-	-	-	-	-
Nasal wash	-	< 5	-	-	-	-	< 5
Other	19 (4.6)	9 (4.0)	18 (4.7)	7 (.5)	< 5	7 (7.5)	22 (4.6)
Missing	-	< 5	-	-	-	-	< 5
**Admitted**	177 (43.4)	99 (44.6)	174 (45.2)	70 (55.1)	52 (53.1)	52 (55.9)	238 (50.2)
**ICU Admission**	44 (10.8)	24 (10.8)	54 (14.0)	19 (12.2)	15 (15.3)	9 (9.7)	55 (11.5)
**Severe COVID-19**	132 (32.3)	66 (29.7)	154 (40.0)	61 (39.1)	40 (40.8)	37 (39.8)	165 (34.5)
**Intubated**	22 (5.4)	9 (4.0)	23 (6.0)	7 (4.5)	< 5	< 5	28 (5.9)
**Steroids**	135 (33.1)	73 (32.8)	137 (35.8)	46 (29.5)	39 (39.8)	39 (41.9)	165 (34.5)
**Death**	23 (5.6)	11 (4.9)	18 (4.7)	< 5	< 5	5 (5.4)	35 (7.3)

ICU=Intensive Care Unit; SARS-CoV-2=Severe acute respiratory syndrome coronavirus 2; NAAT=nucleic acid amplification testing

© 2022 Hohl CM et al. *JAMA Network Open.*

***e*Figure.** Treatments and Outcomes Among Patients by Symptom Duration at the Time of Their First SARS-CoV-2 NAAT Among 96,232 Patients Who Reported a Date of Symptom Onset

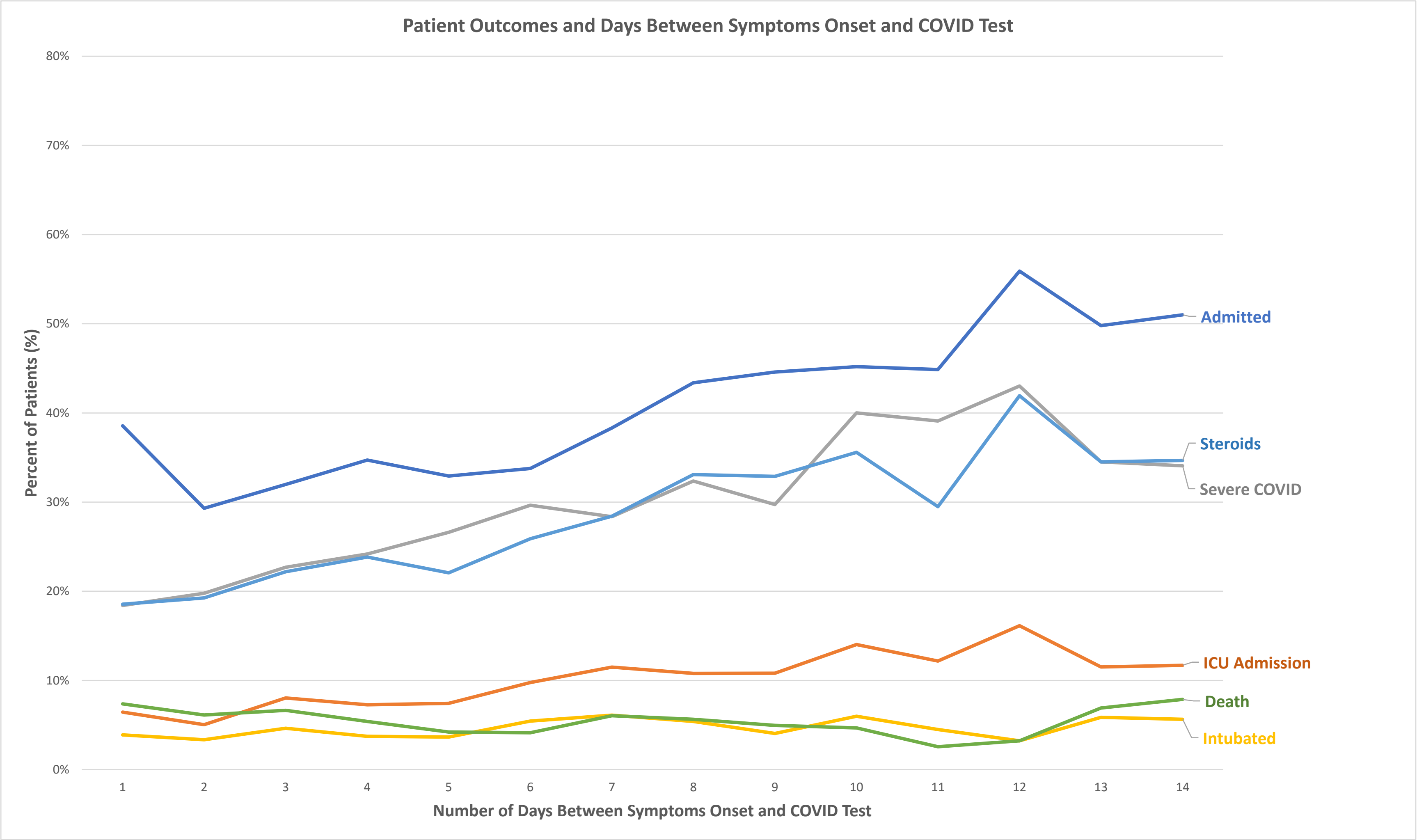

SARS-CoV-2=Severe acute respiratory syndrome coronavirus 2; NAAT=nucleic acid amplification testing

© 2022 Hohl CM et al. *JAMA Network Open.*

eTable 4. Sensitivity of SARS-CoV-2 NAATs, by Symptom Duration Among 12,726 SARS-CoV-2 Positive Patients Who Reported a Date of Symptom Onset

Time	True Positive, No.	False Negative, No.	Number of Tests, No.	Sensitivity, % (95% CI)
<24h	3,238	142	3,380	95.8 (95.0-96.4)
2 days	1,710	41	1,751	97.7 (96.8-98.3)
3 days	1,725	49	1,774	97.2 (96.4-97.9)
4 days	1129	27	1,156	97.7 (96.6-98.5)
5 days	905	28	933	97.0 (95.7-98.0)
6 days	540	27	567	95.2 (93.1-96.8)
7 days	1,492	51	1,543	96.7 (95.7-97.5)
8 days	419	19	438	95.7 (93.3-97.3)
9 days	241	18	259	93.1 (89.2-95.8)
10 days	397	22	419	94.7 (92.2-96.7)
11 days	170	18	188	90.4 (85.3-94.2)
12 days	125	6	131	95.4 (90.3-98.3)
13 days	112	5	117	95.7 (90.3-98.5)
14 days	479	17	496	96.6 (94.6-98.0)

SARS-CoV-2=Severe acute respiratory syndrome coronavirus 2; NAAT=nucleic acid amplification testing

© 2022 Hohl CM et al. *JAMA Network Open.*

eTable 5. Diagnostic Yield by Symptom Duration at the Time of the First SARS-CoV-2 NAAT Among 96,232 Patients Who Reported a Date of Symptom Onset

Time	**COVID Positive**, No.	**COVID Negative**, No.	**Number of Swabs**, No.	**Diagnostic Yield, % (95% CI)**
< 24h	1,686	19033	20,719	8.1 (7.7-8.5)
2 days	1,763	11097	12,860	13.7 (13.1-14.3)
3 days	1,784	9930	11,714	15.2 (14.6-15.9)
4 days	1,171	6276	7,447	15.7 (14.9-16.6)
5 days	952	4286	5,238	18.2 (17.1-19.2)
6 days	595	2708	3,303	18.0 (16.7-19.3)
7 days	1559	6456	8,015	19.5 (18.5-20.3)
8 days	482	2420	2,902	16.6 (15.2-18.0)
9 days	286	1245	1,531	18.7 (16.7-20.6)
10 days	445	1784	2,229	20.0 (18.3-21.6)
11 days	220	1119	1,339	16.4 (14.4-18.4)
12 days	183	899	1,082	16.9 (14.7-19.2)
13 days	160	964	1,124	14.2 (12.2-16.3)
14 days	546	3656	4,202	13.0 (11.9-14.0)

SARS-CoV-2=Severe acute respiratory syndrome coronavirus 2; NAAT=nucleic acid amplification testing

© 2022 Hohl CM et al. *JAMA Network Open.*
