# Supplementary material for: Sensitivity and Diagnostic Yield of the First SARS-CoV-2 Nucleic Acid Amplification Test Performed for Patients Presenting to the Hospital
Source: JAMA Netw Open. 2022 Oct 12;5(10):e2236288. doi: 10.1001/jamanetworkopen.2022.36288 (PMC9557877; doi:10.1001/jamanetworkopen.2022.36288)
Supplement: Supplement 2. — Nonauthor Collaborators. The Canadian COVID-19 Emergency Department Rapid Response Network (CCEDRRN) investigators for the Network of Canadian Emergency Researchers and the Canadian Critical Care Trials Group [file jamanetwopen-e2236288-s002.pdf]

Supplemental Online Content: Nonauthor Collaborators
*First name, last name, and suffix (if applicable) are required and will appear in PubMed.

*Group Name(s): Canadian COVID-19 Emergency Department Rapid Response Network (CCEDRRN) investigators for the Network of Canadian Emergency Researchers and the Canadian Critical Care Trials Group							
***First Name and Middle Initial(s)**	***Last Name**	***Suffix (eg, Jr, III)**	Academic Degrees	Institution	Location (city, state/province, country)	Role or Contribution, eg, chair, principal investigator	Group (if more than 1 Group listed in the byline) and/or Subgroup (eg, Steering Committee)
Ian	Martin		MD	Department of Emergency Medicine, University of British Columbia	Vancouver, British Columbia, Canada	Site Investigator	
John	Taylor		MD	Department of Emergency Medicine, University of British Columbia	Vancouver, British Columbia, Canada	Site Investigator	
Maja	Stachura		MD	Department of Emergency Medicine, University of British Columbia	Vancouver, British Columbia, Canada	Site Investigator	
Frank	Scheuermeyer		MD	Department of Emergency Medicine, University of British Columbia	Vancouver, British Columbia, Canada	Site Investigator	
Daniel	Ting		MD	Department of Emergency Medicine, University of British Columbia	Vancouver, British Columbia, Canada	Site Investigator	
Baljeet	Brar		MD	Department of Emergency Medicine, University of British Columbia	Vancouver, British Columbia, Canada	Site Investigator	
Lee	Graham		MD	Department of Emergency Medicine, University of British Columbia	Vancouver, British Columbia, Canada	Site Investigator	
Ian	Mitchell		MD	Department of Emergency Medicine, University of British Columbia	Vancouver, British Columbia, Canada	Site Investigator	
Andrew	McRae		MD	Emergency Medicine and Community Health Sciences, University of Calgary	Calgary, Alberta, Canada	Site Investigator	

Supplemental Online Content: Nonauthor Collaborators
*First name, last name, and suffix (if applicable) are required and will appear in PubMed.

***First Name and Middle Initial(s)**	***Last Name**	***Suffix (eg, Jr, III)**	Academic Degrees	Institution	Location (city, state/province, country)	Role or Contribution, eg, chair, principal investigator	Group (if more than 1 Group listed in the byline) and/or Subgroup (eg, Steering Committee)
Brian	Row		MD	Department of Emergency Medicine, University of Alberta	Edmonton, Alberta, Canada	Site Investigator	
Jake	Hayward		MD	Department of Emergency Medicine, University of Alberta	Edmonton, Alberta, Canada	Site Investigator	
Jaspreet	Khangura		MD	Department of Emergency Medicine, University of Alberta	Edmonton, Alberta, Canada	Site Investigator	
Phil	Davis		MD	Department of Emergency Medicine, University of Saskatchewan	Saskatoon, Saskatchewan, Canada	Site Investigator	
Michelle	Welsford		MD	Division of Emergency Medicine, McMaster University	Hamilton, Ontario, Canada	Site Investigator	
Robert	Ohle		MD	Department of Emergency Medicine, Northern Ontario School of Medicine	Sudbury, Ontario, Canada	Site Investigator	
Justin	Yan		MD	Department of Emergency Medicine, Western University	London, Ontario, Canada	Site Investigator	
Ivy	Cheng		MD	Department of Emergency Medicine, University of Toronto	Toronto, Ontario, Canada	Site Investigator	
Megan	Landes		MD	Department of Emergency Medicine, University of Toronto	Toronto, Ontario, Canada	Site Investigator	
Rohit	Mohindra		MD	Department of Emergency Medicine, University of Toronto	Toronto, Ontario, Canada	Site Investigator	
Patrick	Archambault		MD	Department of Emergency Medicine, Université Laval	Quebec City, Quebec, Canada	Site Investigator	
Joel	Turner		MD	Department of Emergency Medicine, McGill University	Montreal, Quebec, Canada	Site Investigator	
Lars	Grant		MD	Department of Emergency Medicine, McGill University	Montreal, Quebec, Canada	Site Investigator	
Eric	Mercier		MD	Emergency Deparmtent of CHU de Québec, Université Laval	Quebec City, Quebec, Canada	Site Investigator	

Supplemental Online Content: Nonauthor Collaborators
*First name, last name, and suffix (if applicable) are required and will appear in PubMed.

***First Name and Middle Initial(s)**	***Last Name**	***Suffix (eg, Jr, III)**	Academic Degrees	Institution	Location (city, state/province, country)	Role or Contribution, eg, chair, principal investigator	Group (if more than 1 Group listed in the byline) and/or Subgroup (eg, Steering Committee)
Greg	Clark		MD	Department of Emergency Medicine, McGill University	Montreal, Quebec, Canada	Site Investigator	
Raoul	Daoust		MD	Department of Emergency Medicine, Université de Montréal	Montreal, Quebec, Canada	Site Investigator	
Sébastien	Robert		MD	Emergency Deparmtent of CHU de Québec, Université Laval	Quebec City, Quebec, Canada	Site Investigator	
Kavish	Chandra		MD	Department of Emergency Medicine, Dalhousie University	Saint John, New Brunswick, Canada	Site Investigator	
Patrick	Fok		MD	Department of Emergency Medicine, Dalhousie University	Halifax, Nova Scotia, Canada	Site Investigator	
Hana	Wiemer		MD	Department of Emergency Medicine, Dalhousie University	Halifax, Nova Scotia, Canada	Site Investigator	
Sam	Campbell		MD	Department of Emergency Medicine, Dalhousie University	Halifax, Nova Scotia, Canada	Site Investigator	
Kory	Arsenault		MD	Department of Emergency Medicine, Dalhousie University	Halifax, Nova Scotia, Canada	Site Investigator	
